# A survey assessing the health science students' perception towards online learning at a Saudi Higher Education Institution during COVID-19 pandemic

**DOI:** 10.1016/j.heliyon.2022.e10632

**Published:** 2022-09-16

**Authors:** Nouf Al-Kahtani

**Affiliations:** Health Information Management and Technology Department, College of Public Health, Imam Abdulrahman Bin Faisal University, Dammam, Saudi Arabia

**Keywords:** COVID-19 pandemic, Online learning, Structural equation modeling, Students perception, Survey tool

## Abstract

As the COVID-19 pandemic pushed universities worldwide to shift from traditional to online learning, there is a need to capture the students' perception of online learning using an appropriate tool. Hence, this study explores the appropriateness of the online learning assessment survey (OLAS) model for assessing the students' perception of online learning during the COVID-19 pandemic. It included the undergraduate students (N = 2523) of the selected four health science colleges at Imam Abdulrahman Bin Faisal University (IAU) during 2020–2021. The data was obtained through OLAS using “Google Docs” from 728 students. The structural equation modeling (SEM) analysis revealed that each item showed a significant positive relationship with its respective variable of OLAS. The proposed OLAS model with five variables showed a good fit to assess the students' perception of online learning during the COVID-19 pandemic. Those variables enable the university policy planners to evaluate the students' perception of online learning during the pandemic, thereby supporting them in framing appropriate strategies to improve the quality and success of online learning. Further research is necessary to include all students of various programs offered at Saudi universities to generalize the outcomes. OLAS can include a global item assessing overall students' satisfaction with online learning, and the influence of OLAS variables on the overall students' satisfaction can be evaluated in future studies.

## 1 Introduction

Online learning is coined as “learning experiences in synchronous or asynchronous settings through various applications such as smartphones, laptops, etc., with internet access”. Students can be at any place (independent) to study and interact with faculty and other students in such settings. Online learning is an instrument that could create the teaching and learning process more advanced, student-centered, and flexible (Singh and Thurman, 2019). It is also being treated as a valued instrument for learning, flexibility, cost efficiency, and the option of delivering outstanding education (Almahasees et al., 2021). Moreover, online learning has critical benefits such as self-learning, inexpensive, ease, and suppleness. However, it acts as a barrier to students' engagement in actual class events, and students fail to experience the impact of peer learning. These encounters also influence students' traits and prevent them from taking turns. Besides, online learning has become a slice of the modern world since it utilizes online platforms (Almahasees et al., 2021). Remarkably, the COVID-19 pandemic led to the closing of higher education institutions (HEIs). Such closure exerts a significant burden on those HEIs to manage the unprecedented change from conventional to online learning (Almahasees et al., 2021).

Recently, the quick progression of online learning has made several HEIs dynamically aim for global students to boost them to get online education to save currency. Likewise, Saudi HEIs embark on rapid online education growth (Aziz Ansari et al., 2021). Saudi Arabia is augmenting its educational objectives and dynamically contributing to global educational alterations to meet future cohorts' challenges (Al-Asmari and Rabb Khan, 2014; Asiry, 2017). It has recognized several online HEIs such as “online Islamic university” and “The Saudi electronic university (SEU).” In this state, the COVID-19 pandemic has affected all facets of human life, comprising education. The use of technology is the solitary key to warrant the continuance of education globally. Therefore, a sudden shift to online education occurred in various nations worldwide. The Saudi government is active in applying stringent measures to control the transmission of COVID-19. It directed all schools and HEIs to shut down in March 2020 following the observation of the first COVID-19 confirmed case in the nation (Altuwairesh, 2021). The Ministry of Education (MOE) of Saudi Arabia instructed to conduct online classes to endure a safe and secure learning process. Accordingly, all HEIs, comprising medical schools, were moved to online learning (Tanveer Awan et al., 2020). In adherence to the MOE’s order, HEIs initiated to motivate their students with online education using digital tools, i.e., ZOOM and Microsoft’s Teams application (Aziz Ansari et al., 2021). However, such a sudden shift from traditional to online learning generated the prerequisite for revealing the students' feedback on online learning to improve and sustain the quality of higher education (Aziz Ansari et al., 2021). Consequently, few studies have revealed the students' perception of online learning in the Saudi Arabian context (Linjawi and Alfadda, 2018; Al-Nofaie, 2020; Alkinani, 2021; Altuwairesh, 2021, Aziz Ansari et al., 2021). Notably, Linjawi and Alfadda (2018) revealed the students' perceptions, attitudes, and readiness toward online dental education in a Saudi dental school. It used the questionnaire, which assessed technological access, computer skills, perceived ease of use, utility, social norms, institutional and technical support, and overall readiness. Aziz Ansari et al. (2021) assessed the health sciences students' perception of online teaching and learning in two Saudi HEIs during the COVID-19 lockdown. The targeted students were from the college of medicine, public health, and dentistry. It used only the instrument focusing on the online teaching and learning process, and technical support offered to the students (Aziz Ansari et al., 2021). Altuwairesh (2021) conducted a case study assessing female students' perceptions of online education during the COVID-19 pandemic at a Saudi University. Those undergraduate English language students were administered with a survey measuring the students' perceptions of online education and their challenges, advantages, and disadvantages during online learning. Likewise, Al-Nofaie (2020) led a case study that examined the students' perception of learning using Blackboard during COVID-19 at a Saudi university. The Blackboard readiness survey was administered to the female students of an undergraduate English program. It revealed the encounters and merits of online learning to know the students' learning experiences and recommended appropriate solutions. Lastly, Alkinani (2021) conducted a qualitative study using a structured interview with fifteen undergraduate students of a Saudi public university. It revealed the students' perceptions of online learning during COVID-19. The interview covered the positive and negative experiences of online learning, such as cost-effectiveness, flexibility, availability of the electronic research databases, well-designed online classroom interfaces, lecturer’s delayed feedback, lack of technical support, feeling of isolation, and poorly designed class materials.

On reviewing the literature, those studies revealed the perception of students belonging to the health sciences and English language program towards online learning. However, their instrument failed to measure the students' engagement and interaction. Some of these studies identified that students face the challenges such as the absence of interaction with teachers and students and missing face-to-face/physical interaction while online learning (Al-Nofaie, 2020; Altuwairesh, 2021). Sun and Chen (2016) claimed that the faculty and learners should be deeply involved in building interaction and collaboration, thus forming an active online learning community. Online applications, including Zoom, Collaborate, and Microsoft Teams, possess facets to encourage active learning via student participation and engagement (Aziz Ansari et al., 2021). Moreover, Lei and Zhao (2008) recommended revealing the impact of technology use on student learning outcomes (LOs). However, no studies have uncovered the students' perception of the achievement of LOs of courses through online learning. The perception of students from other health sciences colleges, such as nursing and applied medical sciences, was also uncovered. Therefore, there is a need to develop a survey and reveal the appropriateness of its variables for assessing online learning, especially among health science students. Following the consideration of these gaps, this study developed a new survey named “Online Learning Assessment Survey (OLAS)” (see Appendix). It aimed to determine the appropriateness of the OLAS model for assessing the health science students' perception of online learning during the COVID-19 pandemic. To achieve this study objective, structural equation modeling (SEM) analysis" was implemented using “AMOS (Analysis of Moment Structures) version 5.0.” This study will explore the appropriateness of OLAS variables to assess the health science students' perception of online learning. It will assist higher education administrators in developing appropriate strategies to improve the quality and success of online learning. Furthermore, the study outcomes will contribute to the existing literature on online learning in the COVID-19 pandemic guide for further progression in online learning. Research gaps are exposed, and recommendations are provided for further research to develop more effective online learning at the university level.

To facilitate this study, Imam Abdulrahman Bin Faisal University (IAU) was chosen as it conducted the courses through online platforms and sustained the continuity of the educational process without any delays. It provided enough information and training for its faculty and students to enable effective online teaching and learning.

## 2 Theoretical model

**Figure 1:**
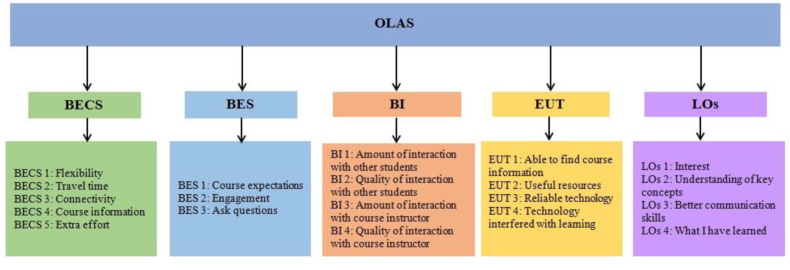
Theoretical model – OLAS.

While reviewing the previous literature, this study framed a theoretical model, “OLAS” (Figure 1), based on five variables relating to online learning. It intended to answer the research question stating whether these variables are significant enough to assess the students' perception of online learning. The five variables of the OLAS model are described as follows:

### 2.1 Better experience for commute students (BECS)

During the COVID-19 pandemic, HEIs worldwide closed their campuses and suddenly moved to online learning, making the students learn remotely and influencing their travel time to HEIs (Crawford et al., 2020; Versteijlen et al., 2021). A previous study reported that online learning had reduced the students' commuting time and travel time to campus (Shim and Lee, 2020). Further, students perceived flexibility as one of the utmost advantages of online learning (Zheng et al., 2021). Such flexibility allows students to plan their coursework during pandemics (CSU Global, 2020). On the other hand, online learning requires additional exertion and concern (Avila, 2020). A recent study also stated that students need more effort to be motivated during online learning events (Almendingen et al., 2021). Several online learning platforms lead to information overload, resulting in overload and raising student stress levels (Al-Kumaim et al., 2021). Students felt challenged during online learning as they had no peers for discussion and getting support in understanding the course contents (Avila, 2020).

### 2.2 Better engage students (BES)

During the COVID-19 pandemic, student engagement in online learning became a significant issue as HEIs shifted to online teaching and learning, using unique platforms to conduct their courses (Dembereldorj, 2021). Student engagement is the level of interest shown by students, how they interact with instructors and peers in the course, and their motivation to learn about the contents (Gray and DiLoreto, 2016). In online learning, it can be analyzed based on the dimensions such as behavioral engagement dealing with the student's participation and interaction, affective engagement describing the students' attitude toward faculty members and peers, and cognitive engagement relating to students' motivation and effort to learn (Salas-Pilco et al., 2022). A study by Deka (2021) identified faculty characteristics, student characteristics, course structure, course content, technology support, and learning environment as factors affecting students' engagement in online learning. Further, student engagement is affected by the elements such as student-instructor interaction, student-content interaction, and student-student interaction. Among these elements, the students perceived that student-instructor interaction is critical in promoting engagement. Most students felt more comfortable asking and responding to questions in online classes (Hollister et al., 2022). Besides, faculty members should communicate the course expectations for guidelines, rubrics, resources, assignments, and end dates to improve students' sustained learning and academic achievement (Gray and DiLoreto, 2016). The assessment of students' engagement permits faculty members to adapt their teaching practices concerning the changes in students' participation, attitude, and drive about their course and educational pursuits (Gray and DiLoreto, 2016).

### 2.3 Better interaction (BI)

A vital issue of online learning is that students feel disconnected from their peers and faculty members (Gray and DiLoreto, 2016). Student-student interaction avoids isolation and boredom by forming an active sense of community (Martin and Bolliger, 2018). In online learning, such interaction is enhanced through peer evaluation, group events, discussions, and chat forums (Hollister et al., 2022). Moreover, student-instructor interaction is an essential element, and it is improved through proper communication, encouraging the student’s active role in discussions, and timely and adequate feedback to students (Hollister et al., 2022). Generally, online learning should permit quality interaction between the student-instructor, student-content, and student-student. Such quality of interaction needs to be confirmed to attain the effectiveness of online learning (Ping, 2011). However, Nieuwoudt (2018) stated that students' online interaction and participation were assessed in quantity compared to quality.

### 2.4 Effective use of technology (EUT)

The effective use of technology is crucial to improving the success of online learning during the COVID-19 pandemic (Kumalawati et al., 2021). Following the closure of HEIs, online teaching and learning were continued through digital tools, such as Blackboard, which delivers a more flexible experience via consistent communication tools (Alshaikh et al., 2021). Students find the valuable features of Blackboard, such as course content, grades, announcements, and assignments by faculty members. However, they felt several technical problems that affect their usefulness during online learning (Alsuhaibani, 2021).

### 2.5 Learning outcomes (LOs)

LOs denote the anticipated outcomes of a course about what a student recognizes, comprehends, and can demonstrate following the course completion (Latif and Subramaniam, 2016). Students valued the online learning environments and stated enhanced understanding of concepts in online courses, knowledge scores, better communication, and student satisfaction (Dailey-Hebert, 2018). Nguyen (2015) stated the positive LOs in online learning, such as student interest in the course material, improved understanding of learning, and enhanced learning represented by test scores. However, several online teaching and learning studies have not emphasized LOs (Ismail, 2021). Hence, assessing the LOS in online learning is significant since the students suddenly shifted to online learning during the COVID-19 pandemic.

## 3 Materials and methods

### 3.1 Survey instrument development

Based on the previous literature, this study developed OLAS, an online questionnaire in “Google Docs” to check the reliability, validity, and model fit of the theoretical model. The OLAS comprised five variables, with 20 items. The five variables of OLAS are: (i) BECS (05 items), (ii) BES (03 items), (iii) BI (04 items), (iv) EUT (04 items), and (v) Los (04 items). The students' level of agreement towards the items in each variable was stated on a five-point Likert scale (“1-strongly disagree”, “2-disagree”, “3-neither agree or disagree”, “4-agree”, “5-strongly agree”). After drafting the survey, it was reviewed by educational experts from the relevant field. Based on their feedback, minor changes were made to the OLAS and made it ready for data collection.

### 3.2 Sample and data collection

The exploratory study design was used to examine the appropriateness of the OLAS model for assessing the health science students' perception of online learning during the COVID-19 pandemic. It covered the entire population of undergraduate students (N = 2523) from the selected four health science colleges of IAU during the academic year 2020–2021. The selected colleges were the College of Medicine (COM), College of Nursing (CON), College of Public Health (CPH), and College of Applied Medical Sciences (CAMS). Among the total population (N = 2523), 800 students were selected using a random sampling method. Those were distributed with OLAS using “Google Docs.”

Subsequently, the participants were requested to provide informed consent, and anonymity and confidentiality were secured before gathering data. Out of 800, 728 completed questionnaires were received, demonstrating a 91% response rate. Among those respondents (n = 728), 11.7% (n = 85) were male, and 88.3% (n = 643) were female. Ethical approval was attained from the Institutional Review Board (IRB) (IRB-2019-03-215) of IAU, Saudi Arabia.

### 3.3 Data analysis

This study applied covariance-based SEM using AMOS software to reveal the appropriateness of the OLAS model containing five variables and 20 items. Recent studies have also used the SEM approach to validate the proposed model (Khan et al., 2019, 2021). Descriptive statistics were used to determine the mean and standard deviation of the responses toward OLAS variables. Moreover, the skewness and kurtosis were applied to test the normality. The reliability was measured through Cronbach’s alpha reliability test and composite reliability (CR). The average variance extracted (AVE) was used for assessing convergent validity. Further, the construct validity was assessed through confirmatory factor analysis. A Pearson correlation analysis was conducted to measure the relationship between OLAS variables. The data analysis was carried out using SPSS. The level of significance was fixed as 5%.

## 4 Results

### 4.1 Descriptive statistics

In this study, all OLAS variables showed their mean score ranging from 3.47 to 3.89. In addition, the skewness and kurtosis values were within the recommended range of ±2, indicating that the data were normal (George, 2011) (Table 1).

**Table 1:** Descriptive statistics of OLAS variables.

Variables	Mean	Standard Deviation	Skewness	Kurtosis
Better experience for commute students (BECS)	3.47	0.720	−0.850	1.191
Better engage students (BES)	3.70	1.025	−0.776	0.376
Better interaction (BI)	3.70	1.048	−0.701	0.205
Effective use of technology (EUT)	3.89	0.934	−0.894	0.924
Learning outcomes (LOs)	3.81	0.986	−0.824	0.691

### 4.2 Reliability and validity of OLAS

While reviewing the reliability of the OLAS, Cronbach’s alpha values for BECS, BES, BI, EUT, and LOs were 0.71, 0.87, 0.94, 0.86, and 0.92, respectively. BI and LOs were observed with Cronbach’s alpha value of >0.90 and were rated as ‘Excellent.’ BES and EUT showed their Cronbach’s alpha (α) value as >0.8. Those were rated as ‘Good.’ Only BECS scored Cronbach’s alpha value of >0.7 and rated it as “Acceptable.” Furthermore, the overall Cronbach's alpha value for all OLAS variables was observed as 0.96, rated as 'Excellent' (George and Mallery, 2003; Jain and Angural, 2017). Furthermore, CR values were higher than the recommended value of 0.70, indicating high reliability (Henseler et al., 2015) (Table 2).

**Table 2:** Reliability and validity of OLAS.

Variables	No. of items	Cronbach’s Alpha (α)	Composite Reliability	Average Variance Extracted
BECS	05	0.71	0.882	0.600
BES	03	0.87	0.743	0.519
BI	04	0.94	0.903	0.699
EUT	04	0.86	0.807	0.521
LOs	04	0.92	0.775	0.517
Overall	20	0.96		

Concerning the validity, the AVE values for all variables were observed to be more than 0.50, denoting no issues with convergent validity (Chin, 1998) (Table 2). Besides, factor analysis measured the KMO value of 0.955 and Bartlett’s test of sphericity value of 13659.632 (*p* < 0.05), which confirmed that the sample was suitable for applying confirmatory factor analysis. Table 3 showed that all OLAS items had a communality value of 0.60 or above, which is recommended by previous researchers (Field, 2009; Mertler and Vannatta, 2010). This finding indicated that the quality of the measurements is satisfactory. The factor loading of all OLAS items was higher than the recommended value of 0.60 (Hair et al., 2011). Five variables were extracted from the original 20 items using the Kaiser criterion and Varimax rotation. These five variables together described 72.543 percent of the variance in health sciences students' perceptions of online learning at a Saudi university (Table 4). These findings reveal that the proposed survey is a reliable and valid tool for assessing online learning among students.

**Table 3:** Common communalities of OLAS.

Item no.	Items	Initial	Extraction
BECS1	This course allowed me to have more flexibility in my personal schedule	1.000	0.689
BECS2	This course allowed me to reduce my total travel time to campus each week	1.000	0.776
BECS3	I felt connected to other students in this course	1.000	0.732
BECS4	I was overwhelmed with information in this course	1.000	0.691
BECS5	This course required extra effort.	1.000	0.693
BES1	The course expectations were clearly communicated.	1.000	0.798
BES2	I was more engaged in this course	1.000	0.690
BES3	I was more likely to ask questions in this course.	1.000	0.687
BI1	The amount of my interaction with other students in this course increased	1.000	0.812
BI2	The quality of my interaction with other students in this course was better.	1.000	0.847
BI3	The amount of my interaction with the instructor in this course increased.	1.000	0.785
BI4	The quality of my interaction with the instructor in this course was better.	1.000	0.823
EUT1	I was able to find course information easily at the Blackboard.	1.000	0.761
EUT2	The resources at the Blackboard were useful.	1.000	0.736
EUT3	The technology used for this course was reliable.	1.000	0.745
EUT4	The technology used in this course interfered with my learning	1.000	0.640
Los 1	Taking this course increased my interest in the material.	1.000	0.701
Los 2	This course improved my understanding of key concepts.	1.000	0.722
Los 3	This course helped me develop better communication skills.	1.000	0.726
Los 4	I had more opportunities in this course to reflect on what I have learned	1.000	0.756

**Table 4:** Factor loading for OLAS.

Items	1	2	3	4	5
This course allowed me to have more flexibility in my personal schedule	0.734				
This course allowed me to reduce my total travel time to campus each week	0.720				
I felt connected to other students in this course	0.788				
I was overwhelmed with information in this course	0.795				
This course required extra effort.	0.831				
The course expectations were clearly communicated.		0.766			
I was more engaged in this course		0.645			
I was more likely to ask questions in this course.		0.689			
The amount of my interaction with other students in this course increased			0.844		
The quality of my interaction with other students in this course was better.			0.859		
The amount of my interaction with the instructor in this course increased.			0.818		
The quality of my interaction with the instructor in this course was better.			0.823		
I was able to find course information easily at the blackboard.				0.808	
The resources at the blackboard were useful.				0.777	
The technology used for this course was reliable.				0.772	
The technology used in this course interfered with my learning				0.610	
Taking this course increased my interest in the material.					0.702
This course improved my understanding of key concepts.					0.620
This course helped me develop better communication skills.					0.704
I had more opportunities in this course to reflect on what I have learned					0.693
Eigen Value	11.82	1.43	1.26	0.73	0.63
Variance explained (%)	59.10	7.158	6.284	3.659	3.168
Total Variance explained (%)	72.543				

### 4.3 Correlation between OLAS variables

Among the OLAS variables, BECS showed a significant moderate positive relationship with BES, BI, EUS, and LOs. Also, BI showed a significant moderate positive relationship with EUS. On the other hand, BES showed a significant strong positive relationship with BI, EUS, and LOs. Likewise, LOs showed a significant strong positive relationship with BI and EUS (Table 5).

**Table 5:** Correlation between OLAS variables.

Variables	BECS	BES	BI	EUS
BES	615∗			
BI	630∗	795∗		
EUS	642∗	760∗	674∗	
LOs	628∗	793∗	770∗	756∗

∗ Significant at 0.05 level.

### 4.4 Structural equation modeling for OLAS

**Figure 2:**
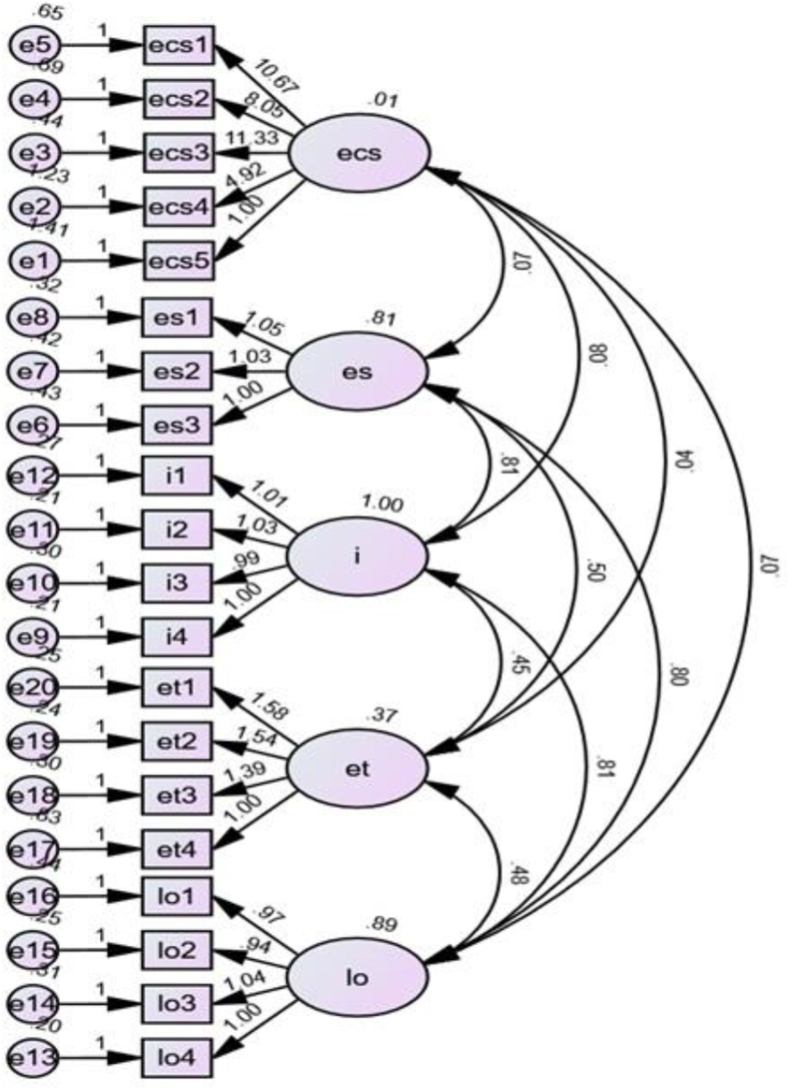
Structural equation modeling (SEM) of OLAS.

SEM analysis was carried out to analyze the appropriateness OLAS model based on the gathered samples. The OLAS model is shown in Figure 2. While evaluating the proposed model, a positive relationship is found between each item with each variable, fluctuating from 0.943 to 1.916 (*p* < 0.05) (Table 6). It is inferred that each item of a variable is positively related to its corresponding variable.

**Table 6:** Regression weights.

Items	Path	Constructs/Variables	Estimate	Standard Error	Critical Ratio	*p*-value
BECS1	<---	BECS	1.000			*p* < 0.05∗
BECS2	<---	BECS	1.046	0.507	11.785	
BECS3	<---	BECS	1.329	0.340	11.787	
BECS4	<---	BECS	1.916	0.796	11.758	
BECS5	<---	BECS	1.671	0.970	11.787	
BES1	<---	BES	1.000			*p* < 0.05∗
BES2	<---	BES	1.033	0.040	26.032	
BES3	<---	BES	1.053	0.039	27.266	
BI1	<---	BI	1.000			*p* < 0.05∗
BI2	<---	BI	1.034	0.028	37.416	
BI3	<---	BI	0.987	0.027	36.942	
BI4	<---	BI	1.008	0.029	34.830	
EUT1	<---	EUT	1.000			*p* < 0.05∗
EUT2	<---	EUT	1.539	.097	15.896	
EUT3	<---	EUT	1.392	.088	15.832	
EUT4	<---	EUT	1.579	.099	15.902	
Los 1	<---	Los	1.000			*p* < 0.05∗
Los 2	<---	Los	.943	.027	34.708	
Los 3	<---	Los	1.035	.030	34.750	
Los 4	<---	Los	.974	.033	29.338	

∗ Significant at 0.05 level.

Furthermore, the outcomes of the model fit indices observed the chi-square value of 882.428 with the following features, i.e., n = 728, degrees of freedom (df) = 160, *p* = 0.000. This chi-square value (882.428) is found to be significant (p < 0.05). However, the recommended chi-square value has to be non-significant (p > 0.05) to accept that the model fits the sample data (Teo et al., 2013). Hence, the chi-square statistics failed to show a good fit for the model. Conversely, the chi-square value is susceptible to an increase in sample size, and the probability level seems significant. Also, it appears to be more as the count of observed variables rises. Thus, a non-significant p-level is rare, though the model might closely fit the observed data. In SEM, the chi-square value cannot be considered the only model fit indicator (Teo et al., 2013). Besides, the relative chi-square/degrees of freedom ratio (CMIN/DF) for this proposed model is observed as 4.765, which is more than the recommended value ranging from 2 to 5 (Paswan, 2009). Other model-fit measures such as “goodness of fit index (GFI)”, “adjusted goodness of fit index (AGFI)”, “comparative fit index (CFI)”, “normed fit index (NFI)”,“incremental fit index (IFI)”, “tucker-lewis index (TLI)”, and “root mean square error of approximation (RMSEA)” were also utilized to evaluate the model. The outcomes of the model fit indices are described in Table 7. The model fit indices except the chi-square value met their respective recommended value, showing that the proposed model is a goodness of fit with the sample data.

**Table 7:** Model fit indices.

Model fit indices	Obtained value	Recommended value
Chi-square	882.428 (p < 0.05)	p > 0.05 (Teo et al., 2013)
Chi-square/degrees of freedom	4.765	2 to 5 (Paswan, 2009)
GFI	0.972	≥0.90 (Byrne, 2001; Kline, 2011)
AGFI	0.961	≥0.80 (Byrne, 2001; Kline, 2011)
CFI	0.973	≥0.90 (Byrne, 2001; Kline, 2011)
NFI	0.964	≥0.90 (Byrne, 2001; Kline, 2011)
IFI	0.974	≥0.95 (Schreiber et al., 2006)
TLI	0.950	≥0.95 (Schreiber et al., 2006)
RMSEA	0.042	<0.05 (Teo et al., 2013)

## 5 Discussion

This study intended to test the appropriateness of the OLAS model for assessing online learning during the COVID-19 pandemic from the students' perspectives. The results demonstrated that OLAS is a reliable and valid tool for evaluating students' perceptions of online learning. Furthermore, the SEM analysis proposed a model using the five variables of the OLAS. It aimed to reveal whether those variables are appropriate for assessing the students' perception of online learning. It is observed that the proposed model is a good fit to measure the students' perception of online learning. There was a positive relationship between each item of a variable and its corresponding variable, recommending that all five variables are potential consequences of online learning. In accord with these results, a recent study investigated the predictors of active online learning in the innovative learning environment using SEM analysis. It is observed that “intelligent interaction”, “personalization”, “real-time feedback”, “perceived ease of use”, and “usefulness of technology” have positively influenced active online learning (Wang et al., 2021). Another study revealed that interaction, motivation, academic integrity, and perceived usefulness influenced the students' perception of online learning (Bui et al., 2021). However, in this study, the variables used in OLAS were BECS, BES, BI, EUT, and LOs. In line with this finding, Muzammil et al. (2020) found that students' engagement positively influenced their satisfaction with online learning. Faculty-student and student-student interaction positively influenced online student engagement, thereby affecting student satisfaction with online learning. Previous studies observed that technology played a role in students' satisfaction with online learning (Kuo et al., 2013; Alqurashi, 2019). Few studies stated that perceived student learning outcome is a crucial forecaster of students' satisfaction with online learning (Marks et al., 2005).

### 5.1 Contributions to the theory

This study offers valuable contributions to the theory that five variables of the OLAS model are significant in assessing the students' perception of online learning. Based on the results, it is observed that BECS is one of the potential variables measuring the students' perception of online learning. This variable deals with the commute students experience in online learning due to the COVID-19 pandemic. Commute students not living in university-owned lodging struggle to make connections outside the classroom. They transport themselves to the university by different means. HEIs should consider those commute students and develop more online services. Linking commute students to virtual communities might support them to feel highly engaged with the university campus (Kretovics, 2015). Earlier studies also discussed commuter students' online learning experience (Galanek and Shulman, 2020; Ranga, 2020). During the pandemic, the most common response of HEIs across the globe was to shut down their campuses and sudden shift to online learning to aid students in enduring their higher education (Crawford et al., 2020). Adopting online learning instead of on-campus offers students a chance to study location independently and might influence their necessity to travel to HEIs. Thus, online learning using the internet reduces the student’s travel time (Versteijlen et al., 2021). Besides, flexibility is one of the essential benefits of online learning. The students mostly learn at their speed and take more time to learn the complex material. Those can retain and recall the information (CSU Global, 2020). Online learning also needs extra effort and responsibility. While studying at home, focusing is challenging as the students have no peers to discuss what is going on and obtain help understanding a subject (Avila, 2020).

Moreover, student engagement is vital for online education since online courses turn students around (McCombs, 2015). It is an effective method to boost online learning (She et al., 2021). It is closely associated with LOs and improves them (Kim and Kim, 2021; She et al., 2021). In online learning, students and faculty interact through internet platforms during their classes (Dembereldorj, 2021). Stimulating interactions through discussions is essential for retaining students' engagement in online learning. In such discussions, faculty motivate the students to raise questions and get a response from their peers (Cao and Duru, 2020). Besides, a clear course objective and expectations are part of course quality and significantly affect students' satisfaction with online learning (Ghaderizefreh and Hoover, 2018). Various researchers have analyzed student engagement during the COVID-19 pandemic (Ali et al., 2021; Dembereldorj, 2021; El-Sayad et al., 2021). An earlier study observed that student engagement is one factor that significantly influenced their success in the online learning setting (Commissiong, 2020; Muzammil et al., 2020). The current study observed that BES is the potential variable for assessing the students' perception of online learning. Martin and Bolliger (2018) also confirmed that student engagement is crucial to students' learning and satisfaction in their learning process; thus, it could improve students' satisfaction. However, Farrell and Brunton (2020) found that active student engagement in online learning was impacted by factors like life load, confidence, course design, peer community, and active instructors. Also, due to inadequate online teaching practice/training, some faculty members using their face-to-face teaching methods online during COVID-19 might negatively influence students' online learning and reduce their engagement (Fazza and Mahgoub, 2021). Consequently, the faculty members should adopt appropriate strategies to develop their teaching practice and monitor students' engagement, thereby achieving and sustaining online learning success.

Besides, online learning platforms permit faculty and students to quickly share their viewpoints through appropriate tools (Wut and Xu, 2021). The participants can use those platforms to discover existing evidence, resolve their issues, and reveal responses based on “student-to-faculty” and “student-to-student” interactions (Wut and Xu, 2021). Furthermore, student-student interaction is vital for students' satisfaction and academic achievement in online learning, allowing students to share their ideas in group tasks (Kurucay and Inan, 2017). A recent study stated that student-to-faculty interaction in online education promotes students' engagement and influences students' learning performance (Sun et al., 2022). It also significantly influences the students' satisfaction (Lin et al., 2017; Kuo et al., 2014). Kuo et al. (2013) revealed that student-to-faculty interaction and student-content interaction were significant forecasters of student satisfaction with online courses, though student-student interaction failed to contribute. An earlier study also observed that student-student interaction was negatively related to student satisfaction with online courses (Arbaugh and Rau, 2007). Muzammil et al. (2020) found that faculty-student and student-student interactions positively influence student engagement in online learning. Also, student engagement positively influenced student satisfaction with online learning. Quality feedback and answers from faculty are equally significant for online learning (Coll et al., 2014). It is recommended that faculty often boost their students to maintain energetic interactions with them and peers in the online classroom environment through various channels (Wut and Xu, 2021). From these statements, the “student-to-faculty” and “student-to-student” interaction is vital for successful online learning, contributing to their student’s learning experiences. This study also found that better interaction between “student-to-faculty” and “student-to-student” is a significant factor in assessing the students' perception of online learning.

On the other hand, using information technology is essential to boost the triumph of online learning during the COVID-19 pandemic (Kumalawati et al., 2021). Further, online teaching and learning were encouraged by the steadiness of the actions through digital tools, such as Blackboard. Accordingly, Saudi HEIs use the most commonly used e-learning platform, i.e., Blackboard. This educational practice provides a more flexible experience through consistent communication tools (Alshaikh et al., 2021). Students were satisfied with Blackboard due to the enhanced content availability, chances for communication and interaction, and ease of use (Al Hassan and Shukri, 2017). Khafaga (2021) revealed that both the “English as a foreign language (EFL)” faculty and students had positive attitudes over the usage of Blackboard during COVID-19. A study conducted in Saudi Arabia found that 67% of students were satisfied with using the Blackboard system in a distance education environment. 57% of students agreed that the Blackboard system enclosed all their educational needs during distance education (Alshaikh et al., 2021). As online learning is encouraged by using the Blackboard system in Saudi HEIs, it is essential to reveal the students' perception of using technology (i.e., Blackboard) during online learning. Accordingly, this study also observed that assessing the effective use of technology from the students' perspectives is vital in online learning. Moreover, technology infrastructure, internet speed, and access affect perceived enjoyment (satisfaction), affecting students' online learning intentions (Maheshwari, 2021). This COVID-19 pandemic led the higher education system to boost the technological infrastructure and their utility among faculty and students community with adequate training and support, thereby overcoming the challenges encountered in the future. Besides, a recent study found that motivation significantly impacted students' perceived ease of use of digital educational technology for online learning during the COVID-19 lockdown. If the students are motivated, their perception of digital educational technology for online learning will be higher (Khan et al., 2022). Thus, HEIs should emphasize the motivation among students toward technology use.

Considering the growing utility of online education, it is significant to evaluate the LOs of students who experience online learning. While compared to traditional face-to-face learning, online learning was highly satisfying and attained better LOs (Morton et al., 2016; Dooley et al., 2018; Green et al., 2018; Riddle and Gier, 2019). Bernard et al. (2014) stated that students performed better with online learning than with traditional learning, and this observation might be due to the enhanced course completion students' motivation and satisfaction. Student motivation, classroom interaction, and course structure affect the students' Los (Baber, 2020). Wells et al. (2008) found that using technology in the educational environment helps attain LOs. Another study stated that student-student interaction, student-content interaction, and student-teacher interaction are vital aspects influencing the LOs in online education (Li et al., 2022). Martin and Bolliger (2018) found that students' engagement is related to positive LOs. During the COVID-19 pandemic, the course structure of online courses should be designed to meet the demands of online learning, thereby improving the students' LO (Martin et al., 2018). A recent study stated that constructive LOs influence students' satisfaction. Perceived LO in online learning is directly proportional to the students' satisfaction during the COVID-19 pandemic (Baber, 2020). Hence, revealing the LOs attained through online learning is crucial based on the course content. This study also observed the LOs as a critical factor in measuring the students' perception of online learning. HEIs must follow uniform guidelines to develop LOs for online courses, which would be helpful in future pandemics.

### 5.2 Practical implications

This study derived the OLAS model to capture students' perceptions of online learning. It would be beneficial to reveal the commuter students' experience and students' engagement and interaction during online learning. In addition, it aids in understanding how effectively they use the technology for online learning and the extent to which the students achieved the LOs of online courses. Such student feedback may prompt instructors and policymakers to focus on OLAS variables, improving online education quality. Further, it supports them in strengthening the online learning environment to overcome the encounters in future pandemics. It also aids them in arranging more training programs to further enhance faculty members' knowledge and skills in handling the online learning environment, thereby sustaining online learning success.

### 5.3 Limitations and recommendations

This study is limited to a single public university covering only health science students. Further research is warranted to cover all students of various programs offered at Saudi Universities to generalize the findings. The survey used in this study can also include a global item measuring overall students' satisfaction with online learning. The influence of five variables of OLAS on the overall students' satisfaction can be measured. As this study focused on revealing the variables that fit to assess the students' perception of online learning, future studies can be conducted with an equal sample size of male and female students to measure the impact of gender on students' perceptions of online learning, especially during the pandemic. Also, factors affecting the students' satisfaction with online learning during the pandemic can be further measured in the Saudi Arabian context.

## 6 Conclusion

This study proposed an OLAS model and revealed its appropriateness in evaluating the students' perception of online learning during the COVID-19 pandemic. Using the SEM analysis, it is concluded that the proposed OLAS model is fit enough to assess the students' perception of online learning. The variables of OLAS include BECS, BES, BI, EUT, and LOs. Those variables enable the policymakers to assess the students' perception of online learning during the pandemic, thereby developing suitable strategies to enhance the quality and success of online learning and enhance their readiness to face future pandemics.

## Declarations

### Author contribution statement

Nouf Al-Kahtani, D. Sc: Conceived and designed the experiments; Performed the experiments; Analyzed and interpreted the data; Contributed reagents, materials, analysis tools or data; Wrote the paper.

### Funding statement

This research did not receive any specific grant from funding agencies in the public, commercial, or not-for-profit sectors.

### Data availability statement

Data will be made available on request.

### Declaration of interest’s statement

The authors declare no conflict of interest.

### Additional information

Supplementary content related to this article has been published online at https://doi.org/10.1016/j.heliyon.2022.e10632.

## Appendix B Supplementary data

Survey-OLAS Survey-OLAS

## References

[bib1] Al Hassan S., Shukri N. (2017). The effect of blended learning in enhancing female students' satisfaction in the Saudi context. Engl. Lang. Teach..

[bib2] Al-Asmari A.M., Rabb Khan M.S. (2014). E-learning in Saudi Arabia: past, present and future. NMEJRE.

[bib3] Al-Kumaim N.H., Alhazmi A.K., Mohammed F., Gazem N.A., Shabbir M.S., Fazea Y. (2021). Exploring the impact of the COVID-19 Pandemic on university students’ learning life: an integrated conceptual motivational model for sustainable and healthy online learning. Sustainability.

[bib4] Al-Nofaie H. (2020). Saudi university students’ perceptions towards virtual education during COVID-19 Pandemic: a case study of language learning via Blackboard. Arab World Engl. J..

[bib5] Ali I., Narayan A.K., Sharma U. (2021). Adapting to COVID-19 disruptions: student engagement in online learning of accounting. Account. Res. J..

[bib6] Alkinani E.A. (2021). Saudi Arabian undergraduate students’ Perceptions of e-learning quality during COVID19 pandemic. IJCSNS Int. J. Comput. Sci. Netw. Secur..

[bib7] Almahasees Z., Mohsen K., Amin M.O. (2021). Faculty’s and students’ perceptions of online learning during COVID-19. Front. Educ..

[bib8] Almendingen K., Morseth M.S., Gjølstad E., Brevik A., Tørris C. (2021). Student’s experiences with online teaching following COVID-19 lockdown: a mixed methods explorative study. PLoS One.

[bib9] Alqurashi E. (2019). Predicting student satisfaction and perceived learning within online learning environments. Distance Educ..

[bib10] Alshaikh K., Maasher S., Bayazed A., Saleem F., Badri S., Fakieh B. (2021). Impact of COVID-19 on the educational process in Saudi Arabia: a technology–organization–environment framework. Sustainability.

[bib11] Alsuhaibani Z. (2021). Saudi EFL Students’ use and perceptions of Blackboard before and during online learning amid COVID-19. Arab World Engl. J. (AWEJ) Spec. Issue CALL.

[bib12] Altuwairesh N. (2021).

[bib13] Arbaugh J.B., Rau B.L. (2007). A study of disciplinary, structural, and behavioral outcomes in online MBS courses. Decis. Sci. J. Innovat. Educ..

[bib14] Asiry M.A. (2017). Dental students’ perceptions of an online learning. Saudi Dent. J..

[bib15] Avila R. (2020). https://thegatewayonline.ca/2020/10/its-time-we-face-it-online-courses-are-more-work/.

[bib16] Aziz Ansari K., Farooqi F., Qadir Khan S., Alhareky M., Trinidad M.A.C., Abidi T., Muzaheed M. (2021). Perception on online teaching and learning among health sciences students in higher education institutions during the COVID-19 lockdown-ways to improve teaching and learning in Saudi colleges and universities. F1000Research..

[bib17] Baber H. (2020). Determinants of students’ perceived learning outcome and satisfaction in online learning during the pandemic of COVID19. J. Educ. e-Learn. Res..

[bib18] Bernard R.M., Borokhovski E., Schmid R.F., Tamim R.M., Abrami P.C. (2014). A meta-analysis of blended learning and technology use in higher education: from the general to the applied. J. Comput. High Educ..

[bib19] Bui D.S., Nguyen H.T., Nguyen V.T., Nguyen V.T., Pham L.K.T. (2021). Students’ perception of online learning during Covid-19 pandemic in Vietnam - a case study on the students of banking university of Ho chi Minh City. Nat. Volatiles & Essent. Oils..

[bib20] Byrne B.M. (2001).

[bib21] Cao E., Duru M. (2020). https://www.povertyactionlab.org/blog/3-26-20/how-keep-students-engaged-online-learning.

[bib22] Chin W.W. (1998). The partial least squares approach to structural equation modelling. Modern Methods Bus. Res..

[bib23] Coll C., Rochera M.J., de Gispert I. (2014). Supporting online collaborative learning in small groups: teacher feedback on learning content, academic task and social participation. Comp. Educ..

[bib24] Commissiong M.A. (2020). https://scholarworks.waldenu.edu/cgi/viewcontent.cgi?article=9794&context=dissertations.

[bib25] Crawford J., Butler-Henderson K., Rudolph J., Malkawi B., Glowatz M., Burton R., Magni P.A., Lam S. (2020). COVID-19: 20 countries’ higher education intra-period digital pedagogy responses. J. Appl. Learn. Teach..

[bib26] Dailey-Hebert A. (2018). Maximizing interactivity in online learning: moving beyond discussion boards. J. Educ. Online.

[bib27] Deka P.K. (2021). Factors influencing student engagement in online learning during the COVID-19 pandemic period in India. J. Manag. Pract..

[bib28] Dembereldorj Z. (2021). Exploring online student engagement during COVID-19 pandemic in Mongolia. Int. J. High. Educ..

[bib29] Dooley L.M., Frankland S., Boller E., Tudor E. (2018). Implementing the flipped classroom in a veterinary pre-clinical science course: student engagement, performance, and satisfaction. J. Vet. Med. Educ..

[bib30] El-Sayad G., Md Saad N.H., Thurasamy R. (2021). How higher education students in Egypt perceived online learning engagement and satisfaction during the COVID-19 pandemic. J. Comput. Educ*.*.

[bib31] Farrell O., Brunton J. (2020). A balancing act: a window into online student engagement experiences. Int. J. Educ. Technol. High. Educ..

[bib32] Fazza H., Mahgoub M. (2021). Student engagement in online and blended learning in a higher education institution in the Middle East: challenges and solutions. Stud. Technol. Enhanc. Learn..

[bib33] Field A. (2009).

[bib34] Galanek J., Shulman B. (2020). https://er.educause.edu/blogs/2020/5/commuter-students-learning-environment-preferences#:~:text=In%202018%2C%203%2065%25%20of,%25%20of%20on%2Dcampus%20students.

[bib35] George D. (2011).

[bib36] George D., Mallery P. (2003).

[bib37] Ghaderizefreh S., Hoover M.L. (2018). Student satisfaction with online learning in a blended course. Int. J. Dig. Soc. (IJDS).

[bib38] CSU Global (2020). https://csuglobal.edu/blog/what-makes-an-online-learning-experience-effective.

[bib39] Gray J.A., DiLoreto M. (2016). The effects of student engagement, student satisfaction, and perceived learning in online learning environments. Int. J. Educ. Leader. Preparat..

[bib40] Green R.A., Whitburn L.Y., Zacharias A., Byrne G., Hughes D.L. (2018). The relationship between student engagement with online content and achievement in a blended learning anatomy course. Anat. Sci. Educ..

[bib41] Hair J.F., Ringle C.M., Sarstedt M. (2011). PLS-SEM: indeed a silver bullet. J. Market. Theor. Pract..

[bib42] Henseler J., Ringle C.M., Sarstedt M. (2015). A new criterion for assessing discriminant validity in variance-based structural equation modeling. J. Acad. Market. Sci..

[bib43] Hollister B., Nair P., Hill-Lindsay S., Chukoskie L. (2022). Engagement in online learning: student attitudes and behavior during COVID-19. Front. Educ..

[bib44] Ismail S. (2021). https://scholar.uwindsor.ca/major-papers/173.

[bib45] Jain S., Angural V. (2017). Use of Cronbach's alpha in dental research. Medrech.

[bib46] Khafaga A.F. (2021). The perception of Blackboard collaborate-based instruction by EFL majors/teachers amid COVID-19: a case study of Saudi universities. J. Lang. Linguist. Stud..

[bib47] Khan M., Lee H.Y., Bae J.H. (2019). The role of transparency in humanitarian logistics. Sustainability.

[bib48] Khan M., Imtiaz S., Parvaiz G.S., Hussain A., Bae J. (2021). Integration of internet-of-things with blockchain technology to enhance humanitarian logistics performance. IEEE Access.

[bib49] Khan M., Parvaiz G.S., Bashir N., Imtiaz S., Bae J. (2022). Students’ key determinant structure towards educational technology acceptance at universities, during COVID 19 lockdown: Pakistani perspective. Cogent Educ..

[bib50] Kim S., Kim D.-J. (2021). Structural relationship of key factors for student satisfaction and achievement in asynchronous online learning. Sustainability.

[bib51] Kline R.B. (2011).

[bib52] Kretovics M. (2015). Commuter students, online services, and online communities. N. Dir. Student Serv..

[bib53] Kumalawati R., Murliawan K.H., Yuliarti A., Kartika N.Y., Noermelani E. (2021). Utilization of information technology for learning in COVID-19 disaster conditions. IOP Conf. Ser. Earth Environ. Sci..

[bib54] Kuo Y.-C., Walker A.E., Belland B.R., Schroder K.E.E. (2013). A predictive study of student satisfaction in online education programs. Int. Rev. Res. Open. Dis..

[bib55] Kuo Y.C., Walker A.E., Schroder K.E.E., Belland B.R. (2014). Interaction, internet self-efficacy, and self-regulated learning as predictors of student satisfaction in online education courses. Internet High Educ..

[bib56] Kurucay M., Inan F.A. (2017). Examining the effects of learner-learner interactions on satisfaction and learning in an online undergraduate course. Comput. Educ..

[bib57] Latif L.A., Subramaniam T.T. (2016). Students’ learning outcomes in online courses: continual quality improvement. Pan-Commonwealth Forum.

[bib58] Lei J., Zhao Y. (2008). One-to-one computing: what does it bring to schools?. J. Educ. Comput. Res..

[bib59] Li X., Lin X., Zhang F., Tian Y. (2022). What matters in online education: exploring the impacts of instructional interactions on learning outcomes. Front. Psychol..

[bib60] Lin C.H., Zheng B., Zhang Y. (2017). Interactions and learning outcomes in online language courses. Br. J. Educ. Technol..

[bib61] Linjawi A.I., Alfadda L.S. (2018). Students' perception, attitudes, and readiness toward online learning in dental education in Saudi Arabia: a cohort study. Adv. Med. Educ. Pract..

[bib62] Maheshwari G. (2021). Factors affecting students’ intentions to undertake online learning: an empirical study in Vietnam. Educ. Inf. Technol..

[bib63] Marks R.B., Sibley S.D., Arbaugh J.B. (2005). A structural equation model of predictors for effective online learning. J. Manag. Educ..

[bib64] Martin F., Bolliger D.U. (2018). Engagement matters: student perceptions on the importance of engagement strategies in the online learning environment. Online Learn..

[bib65] Martin F., Wang C., Sadaf A. (2018). Student perception of helpfulness of facilitation strategies that enhance instructor presence, connectedness, engagement and learning in online courses. Internet High. Educ. Next.

[bib66] McCombs B. (2015). Learner-Centered online instruction. New Dir. Teach. Learn..

[bib67] Mertler C.A., Vannatta R.A. (2010).

[bib68] Morton C.E., Saleh S.N., Smith S.F., Hemani A., Ameen A., Bennie T.D., Toro-Troconis M. (2016). Blended learning: how can we optimise undergraduate student engagement?. BMC Med. Educ..

[bib69] Muzammil M., Sutawijaya A., Harsasi M. (2020). Investigating student satisfaction in online learning: the role of student interaction and engagement in distance learning university. Turk. Online J. Dist. Educ..

[bib70] Nguyen T. (2015). The effectiveness of online learning: beyond no significant difference and future horizons. Merlot J. Online Learn. Teach..

[bib71] Nieuwoudt J. (2018). Exploring online interaction and online learner participation in an online science subject through the lens of the interaction equivalence theorem. Student Success.

[bib72] Paswan A. (2009).

[bib73] Ping T.A. (2011). Students' interaction in the online learning management systems: a comparative study of undergraduate and postgraduate courses. Asian Assoc. Open Univ. J..

[bib74] Ranga S. (2020). Online engagement of commuter students in a general chemistry course during COVID-19. J. Chem. Educ..

[bib75] Riddle E., Gier E. (2019). Flipped classroom improves student engagement, student performance, and sense of community in a nutritional sciences course (P07-007-19). Cur. Dev. Nutr..

[bib76] Salas-Pilco S.Z., Yang Y., Zhang Z. (2022). Student engagement in online learning in Latin American higher education during the COVID-19 pandemic: a systematic review. Br. J. Educ. Technol..

[bib77] Schreiber J.B., Nora A., Stage F.K., Barlow E.A., King J. (2006). Reporting structural equation modeling and confirmatory factor analysis results: a review. J. Educ. Res..

[bib78] She L., Ma L., Jan A., Sharif Nia H., Rahmatpour P. (2021). Online learning satisfaction during COVID-19 pandemic among Chinese university students: the serial mediation model. Front. Psychol..

[bib79] Shim T.E., Lee S.Y. (2020). College students' experience of emergency remote teaching due to COVID-19. Child. Youth Serv. Rev..

[bib80] Singh V., Thurman A. (2019). How many ways can we define online learning? a systematic literature review of definitions of online learning (1988-2018). Am. J. Dist. Educ..

[bib81] Sun A., Chen X. (2016). Online education and its effective practice: a research review. J. Inf. Technol. Educ..

[bib82] Sun H.-L., Sun T., Sha F.-Y., Gu X.-Y., Hou X.-R., Zhu F.-Y., Fang P.-T. (2022). The influence of teacher–student interaction on the effects of online learning: based on a serial mediating model. Front. Psychol..

[bib83] Tanveer Awan M., Bhaumik A., Hassan S., UlHaq I. (2020). COVID-19 pandemic, outbreak educational sector and students online learning in Saudi Arabia. J. Enterpren. Educ..

[bib85] Teo T., Tsai L.T., Yang C.C., Khine M.S. (2013). Application of Structural Equation Modeling in Educational Research and Practice. Contemporary Approaches to Research in Learning Innovations.

[bib86] Versteijlen M., van Wee B., Wals A. (2021). Exploring sustainable student travel behaviour in The Netherlands: balancing online and on-campus learning. Int. J. Sustain. High..

[bib87] Wang S., Shi G., Lu M., Lin R., Yang J. (2021). Determinants of active online learning in the smart learning environment: an empirical study with PLS-SEM. Sustainability.

[bib88] Wells P., de Lange P., Fieger P. (2008). Integrating a virtual learning environment into a second-year accounting course: determinants of overall student perception. Account. Finance.

[bib89] Wut T.-M., Xu J. (2021). Person-to-person interactions in online classroom settings under the impact of COVID-19: a social presence theory perspective. Asia Pac. Educ. Rev..

[bib90] Zheng M., Bender D., Lyon C. (2021). Online learning during COVID-19 produced equivalent or better student course performance as compared with pre-pandemic: empirical evidence from a school-wide comparative study. BMC Med. Educ..

