# Supplementary material for: A survey assessing the health science students' perception towards online learning at a Saudi Higher Education Institution during COVID-19 pandemic
Source: Heliyon. 2022 Sep 16;8(9):e10632. doi: 10.1016/j.heliyon.2022.e10632 (PMC9477607; doi:10.1016/j.heliyon.2022.e10632)
Supplement: Survey-OLAS [file mmc1.docx]

**ONLINE LEARNING ASSESSMENT SURVEY FOR STUDENTS**

**I. Demographic Data:**

**Gender:** Male Female **Age:** ………………………

**College:** College of Medicine College of Applied Medical Sciences

College of Nursing College of Public Health

**Department:** ………………………… **Course Title:** ………………………

**Year of Study:**  1^st^ Year   2^nd^ Year 3^rd^ Year 4^th^ Year 5^th^ Year 6^th^ Year

**II. Students’ Perception towards Online Learning**

***Please select any one response to each question. Be assured that your responses will be kept confidential***

| **S. No.** | **Items** | **Strongly Disagree**  **(1)** | **Disagree**  **(2)** | **Neither agree nor disagree**  **(3)** | **Agree**  **(4)** | **Strongly Agree**  **(5)** |
| --- | --- | --- | --- | --- | --- | --- |
| **I. Better experience for commute student (BECS)** | | | | | | |
| 1 | This course allowed me to have more flexibility in my personal schedule. |  |  |  |  |  |
| 2 | This course allowed me to reduce my total travel time to campus each week |  |  |  |  |  |
| 3 | I felt connected to other students in this course. |  |  |  |  |  |
| 4 | I was overwhelmed with information in this course |  |  |  |  |  |
| 5 | This course required extra effort. |  |  |  |  |  |
| **II. Better engage students (BES)** | | | | | | |
| 6 | The course expectations were clearly communicated. |  |  |  |  |  |
| 7 | I was more engaged in this course |  |  |  |  |  |
| 8 | I was more likely to ask questions in this course. |  |  |  |  |  |
| **III. Better Interaction (BI)** | | | | | | |
| 9 | The amount of my interaction with other students in this course increased |  |  |  |  |  |
| 10 | The quality of my interaction with other students in this course was better. |  |  |  |  |  |
| 11 | The amount of my interaction with the instructor in this course increased. |  |  |  |  |  |
| 12 | The quality of my interaction with the instructor in this course was better. |  |  |  |  |  |
| **IV. Effective use of technology (EUT)** | | | | | | |
| 13 | I was able to find course information easily at the blackboard. |  |  |  |  |  |
| 14 | The resources at the blackboard were useful. |  |  |  |  |  |
| 15 | The technology used for this course was reliable. |  |  |  |  |  |
| 16 | The technology used in this course interfered with my learning |  |  |  |  |  |
| **V. Learning outcomes (LOs)** | | | | | | |
| 17 | Taking this course increased my interest in the material. |  |  |  |  |  |
| 18 | This course improved my understanding of key concepts. |  |  |  |  |  |
| 19 | This course helped me develop better communication skills. |  |  |  |  |  |
| 20 | I had more opportunities in this course to reflect on what I have learned. |  |  |  |  |  |

**Thank You**
